# The Influence of the Prolactins on the Development of the Uterus in Neonatal Mice

**DOI:** 10.3389/fvets.2022.818827

**Published:** 2022-02-17

**Authors:** Jinwen Kang, Yingnan Liu, Yu Zhang, Wankun Yan, Yao Wu, Renwei Su

**Affiliations:** College of Veterinary Medicine, South China Agricultural University, Guangzhou, China

**Keywords:** mice, uterine glands, prolactin family, progesterone, proliferation

## Abstract

The endometrial gland is one of the most important components of the mammalian uterus. However, few studies have been conducted on the regulatory mechanisms of adenogenesis during the development of endometrium. In the present study, we detected the genes expression of 35 different prolactin family members (PRLs) together with the prolactin receptor (PRL-R) in the endometrium of neonatal mice along with the adenogenesis process, to address which prolactin-like genes play a key role during gland development in mice. We found that: (1) The expression of *Prl1a1, Prl3d1, Prl5a1, Prl7a1, Prl7a2, Prl7d1, Prl8a6, Prl8a8*, and *Prl8a9* genes were significantly increased along with the development of uterine glands. *Prl7c1* and *Prl8a1* were observably up-regulated on Postnatal day 5 (PND5) when the uterine glandular bud invagination begins. *Prl3a1, Prl3b1*, and *Prl7b1* suddenly increased significantly on PND9. But, *Prl3c1* and *Prl8a2* were markedly down-regulated on PND5 and the expression of *Prl6a1* and *Prlr* were stable extremely. (2) After continuous injection of Progesterone (P4), a well-known method to suppress the endometrial adenogenesis, the expression of *Prl1a1, Prl3d1, Prl5a1, Prl7a1, Prl7a2, Prl7d1, Prl8a6, Prl8a8, Prl8a9*, and *Prlr* were suppressed on PND7. And on PND9, *Prl1a1, Prl3d1, Prl8a6, Prl8a8*, and *Prl8a9* were significantly inhibited. (3) Further analysis of the epithelial and stroma showed that these PRLs were mainly expressed in the endometrial stroma of neonatal mice. Our results indicate that multiple PRLs are involved in uterine development and endometrial adenogenesis. Continued progesterone therapy may alter the expression pattern of these PRLs in endometrial stromal cells, thereby altering the interaction and communication between stroma and epithelium, and ultimately leading to complete suppression of endometrial adenogenesis.

## Introduction

The uterus is a key reproductive organ for female mammals to reproduce offspring. In mammals, the uterus develops from the accessory middle renal tube, or Mueller's tube, which gives rise to the funnel, fallopian tube, uterus, cervix, and vaginal vestibule (1). Common morphogenetic events during uterine development in all mammals include tissue and stratification of the endometrial interstitium, differentiation, and growth of myometrium, and coordinated development of the endometrial gland (2, 3). A series of secretions synthesized by the uterine glands, named “uterine milk,” are proved to be important for the establishment of mammalian pregnancy, evident from genetically engineered rodents (4). Leukemia inhibitory factor (LIF) and calcitonin are two important secretions of the uterine glands that are essential for the establishment of uterine receptivity and embryo implantation (5). In mice, LIF expression is induced by estrogen on day 4 of gestation and is secreted into the endometrial cavity, regulating embryo implantation and decidualization. The knockout of LIF in the mouse uterus leads to the failure of embryo implantation, and further results in pregnancy failure (6). Calcitonin, a peptide hormone that regulates calcium homeostasis, is secreted by the endometrial glandular epithelium on day 2 of gestation in mice and peaks the day before implantation (Day 4) to regulate embryo implantation (7). Calcitonin also modulates the expression of certain genes in the endometrium, including down-regulating the E-cadherin expression in rodent uterine epithelium (8).

Histologically, the endometrium is composed of two epithelial cell types, the luminal epithelium (LE) and glandular epithelium (GE). Uterine glands are found in the uterus of all mammals. At birth, the mouse uterus has no uterine glands, consisting only of simple epithelium and undifferentiated stroma. In the process of uterine gland development, firstly, some endometrial epithelial cells gradually change from cube cells to cone cells, and the epithelial cells sag to form glandular buds at Postnatal day 5 (PND5) (9). Then, the glandular epithelial cells proliferate and sink deeper into the stroma, eventually forming tubular glands at PND9. Finally, the single tubular gland traverses, curls, and branches throughout the uterine stroma until the apex reaches the inner circular layer of the myometrium, eventually forming a coiled gland (10). Uterine morphogenesis in neonatal ovine, porcine, and rodents are associated with the interactions between epithelium and stroma (11).

Prolactin, originally named for its ability to stimulate lactation behavior, is a protein hormone secreted by the anterior pituitary gland. Afterward, scientists have suggested that prolactin is not only synthesized by the anterior pituitary, (12) but also by the central nervous system, immune system, and uterus, especially decidual (13) and placenta (14, 15). Its function is not limited to reproduction (16). Transcript variations of prolactin are found in many species of mammals, including goats, pigs, cattle, rats, and mice. The variation of prolactin may result from selective splicing of transcripts, proteolytic cleavage, or post-translational modification of amino acid chains (17, 18). In humans, there is only one member in the prolactin family, the PRL (19, 20). But in other species, such as mice, rats, and cattle, the locus has undergone species-specific amplification (21). In mice, the PRL locus spans 1 Mb on chromosome 13 and consists of 35 subtypes, including 23 known genes and 12 genes that have not yet been reported (19, 22).

Prolactin is highly expressed in mammalian female reproductive organs, such as uterine decidual tissue and placenta, indicating that prolactin plays a key role in the reproductive process in the uterus (20). Previous studies have shown that the ineffective mutation of the PRL receptor gene leads to female infertility (23). *Prl1a1* knockout results in female mice infertility (24). *Prl7d1*^−/−^ knockout results in smaller litter size (25). In adult mice, rabbits, and pigs, hyperprolactinemia stimulates uterine glandular hypertrophy (26). High prolactin increases the number and density of glands in the uterus of neonatal ovine (27). In newborn ewes, serum PRL levels are high at birth, increase between PND 1 and 14, and then begin to decline at PND 56, a pattern positively correlated with the occurrence of developing endometrial gland proliferation (28). However, to our best knowledge, none of the single PRL family gene knockout mice show an adenogenesis phenotype. Considering a large number of PRL family members in mice, it is not a surprise that a single-gene knockout has no significant effect on endometrial adenogenesis.

To investigate the association between different prolactin family members and endometrial adenogenesis in neonatal mice, we conducted this in-depth study in animal models. The results show that many prolactin family members, including *Prl1a1, Prl3d1, Prl5a1, Prl7a1, Prl7a2, Prl7d1, Prl8a6, Prl8a8*, and *Prl8a9* are involved in endometrial adenogenesis. Prolactins play an irreplaceable role in glandular genesis.

## Materials and Methods

### Animals

**Figure 1:**
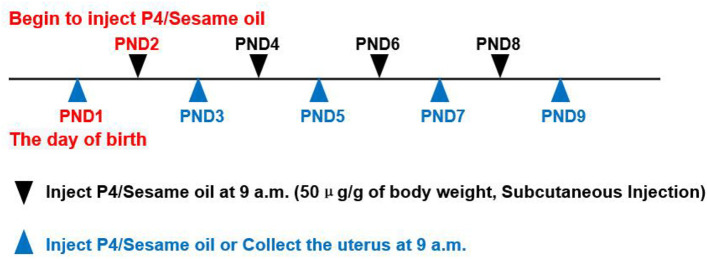
Progesterone(P4)/Sesame oil injection method and the time points of collecting uterus. PND1 = Postnatal Day 1, can deduce the rest from this. P4 = Progesterone. Use a microsyringe (HAMILTON,702 RN,25μl, P/N:7636-01/00) for injection.

All animal procedures were approved by the Institutional Animal Care and Use Committees of South China Agricultural University. C57BL/6J mice were raised in a specific pathogen-free barrier facility at the Animal Center of South China Agricultural University, under 12 h of light-dark cycle from 7 a.m. to 7 p.m., with free reach to food and water. Female mice at 8 weeks of age were bred with fertile male mice. The day of birth of the neonatal mice were recorded as Postnatal Day 1 (PND1). The pups were randomly divided into two groups, the treatment group which received 50 μg/g of body weight progesterone in sesame oil subcutaneously every other day from PND2 till sacrificed to inhibit the generation of endometrial glands of neonatal mice, and the mice received vehicle only served as the control group. At 9 a.m., uteri were collected from the neonatal mice on PND3, PND5, PND7, and PND9 (N≥4 per group). After removing the mesometrium, the uterus was snap-frozen in liquid nitrogen for RNA isolation, fixed in 4% paraformaldehyde (PFA) for histological analysis, or directly used for epithelium-stroma-separation (Figure 1).

### Separation of Endometrial Epithelium and Stroma

The separation method of endometrial epithelial cells and stromal cells is improved and summarized by referring to previous methods (29). The uterus was washed three times in 1xPBS and then incubated in digestive solution for endometrial epithelial separation at 4°C for 1.5 ~ 2.0 h. The Eppendorf tube was inverted up and down 5 ~ 10 times at 0.5 h intervals. The uterus was then transferred to 1xPBS supplemented with 10% FBS to terminate the digestion and then washed 3 times in 1 × PBS. At last, the tubular endometrial epithelial cells were flashed out using a syringe with a 25G needle. The pure epithelium and the remaining stromal tissue were collected for RNA isolation. By real-time PCR, epithelial cell marker *Calbindin 1* (*Calb1*) and stromal cell marker *Homeobox A10* (*Hoxa10*) and *Vimentin* (*Vim*) were detected for purity analysis (Supplementary Figure S1). The digestive solution for endometrial epithelial separation contains the following components (5 mL): 3.5 mL 1xHBSS, 0.5 mL 9.5%Trypsin, 0.5 mL 0.5%Trypsin/EDTA, 0.5 mL 60g/L DispaseII, 200 μL 1IU/μL DnaseI, 50 μl 10xPenicillin Streptomycin.

### RNA Extraction, RT-PCR, and qRT-PCR Analyses

Total RNA was extracted from tissues using TRIzol reagent according to the manufacturer‘s introduction. The RNAs were then reverse transcripted into cDNA using a HiScript cDNA synthesis kit (Vazyme). qPCR was performed using an SYBR qPCR Master Mix kit (Vazyme) following the manufacturer's protocol on the BIORAD-CFX96 Real-Time System (Bio-Rad, Hercules, CA, USA). The data were normalized by mouse *Rpl19* and analyzed using the ΔΔCt method. All qPCR primers of mice were listed in Table 1.

**Table 1:** Characteristics of primers and quantitative real-time PCR reaction conditions used in the study.

**Gene**	**Primer sequences**	**Access number**	**Annealing temperature**	**Product size**
*Prl1a1*	F:5′-CAGAAAGCAGGGACACTCCTCC-3′	NM_011164.2	62°C	125
	R:5′-ACACGGTCAAACAGCTCTCGG-3′			
*Prl2a1*	F:5′-CGCTGCTTTGGTTCCTTTGA-3′ R:5′- TGGCTTGACTCCCTGGTTTT-3′	NM_019991.1	59°C	190
*Prl2b1*	F:5′- TTCTTGGACGAACGCTCTGA-3′ R:5′- CAGGCAGAGTAGGTCGCAAT-3′	NM_025532.3	59°C	177
*Prl2c1*	F:5′- CAAGCCAGGCTCACACACTA-3′ R:5′- TGAGGGCATTAACCCCGTTC-3′	NM_031191.2	60°C	190
*Prl3a1*	F:5′- AGGAGCCTGCAAGACCATAC-3′ R:5′- ATCTGCCAGTCCCATCCAAG-3′	NM_025896.2	59°C	145
*Prl3b1*	F:5′- CCAACGTGTGATTGTGGTGTC-3′ R:5′- TGTGGCAGGGGCTTAACATC-3′	NM_008865.4	60°C	122
*Prl3c1*	F:5′- ATTGACTCAAGCACGCACCT-3′ R:5′- GTGACGAGAAGAGGAAAGCAGA-3′	NM_013766.2	60°C	139
*Prl3d1*	F:5′-GACTACCCTGCTTGGTCTGG-3′	NM_001205322.1	60°C	145
	R:5′-CAACTCGGCACCTCAAGACT-3′'			
*Prl4a1*	F:5′- CCCAGAGCATGAATCACCGA-3′ R:5′- GTTGACCCAGGCTTGCAGTA-3′	NM_011165.4	60°C	200
*Prl5a1*	F:5′-ACACTTCATCCCGCTCTGTC-3′	NM_023746.4	60°C	110
	R:5′-CAGAAGGTTTTTCCAGGCAGC-3′			
*Prl6a1*	F:5′- GTCAACCTTGCTTCTCAGGGA-3′ R:5′- TGGTGACGTGATCAAGCAGG-3′	NM_011166.2	60°C	144
*Prl7a1*	F:5′-CTGCTGGTGGTGTCAAACCT-3′	NM_001164058.1	60°C	112
	R:5′-TGGCATGATCCAACAGTCCC-3′			
*Prl7a2*	F:5′-CTGCTGGTGGTGGTGTCAAG-3′	NM_011168.4	58°C	158
	R:5′-CGCAGTTCCATGTTGAGGTT-3′			
*Prl7b1*	F:5′-CGAAATGCTCACCGAAGACCT-3′	NM_029355.2	60°C	200
	R:5′-ATTTGAGTGGCAAAGTGTGGC-3′			
*Prl7c1*	F:5′- CAGCTGCCACACATTTTCCC-3′ R:5′- CCTGATGGCGTTGGCTCTTA-3′	NM_026206.3	60°C	193
*Prl7d1*	F:5′-GGGACTCTCCTGATGCTGTTG-3′	NM_011120.2	60°C	136
	R:5′-TCTCTCCAGAAAGTACAGTGGC-3′			
*Prl8a2*	F:5′- AACCTCACTTCTCAGGGGCA-3′ R:5′- GAGCAGCCATTCTCTCCTGTT-3′	NM_010088.2	60°C	106
*Prl8a6*	F:5′- ACTCCTGCTGCTACTGTTGTC-3′ R:5′- TGTTTCCATCCGTGGGTTCC-3′	NM_001271378.1	60°C	106
*Prl8a8*	F:5′- ATGCACTCCTGCTGCTACTG-3′ R:5′- TTTCCATCCGTGGGTTCCAG-3′	NM_001311125.1	60°C	108
*Prl8a9*	F:5′-TGCTGCTGGTGGTATCAAAC-3′	NM_023332.4	59°C	139
	R:5′-AAGGGTTCCAGCTGTCTGC-3′			
*Prlr-L*	F: 5′- GTGCAAGAAGGGGCCAAAAG-3′	NM_011169.5	60°C	80
	R: 5′- ACAATGGGGCCTTTCTCCTG-3′			
*Prlr-R*	F: 5′-AAGCCAGACCATGGATACTGGAG-3′ F:5′-TTGTATTTGCTTGGAGAGCCAGT-3′	NM_001253781.1	60°C	254
*Pgr*	F:5′-GCAATGGAAGGGCAGCATAAC-3′	NM_008829.2	60°C	135
	R:5′-CTTACGACCTCCAAGGACCA-3′			
*Esr1*	F:5′-GGAAGCTCCTGTTTGCTCCT-3′	NM_007956.5	60°C	106
	R:5′-AACCGACTTGACGTAGCCAG-3′			
*Ki67*	F:5′-CCAGCTGCCTGTAGTGTCAA-3′	NM_001081117.2	60°C	138
	R:5′-CCATGTCTCAGCCTCACAGG-3′			
*Foxa2*	F:5′-TATGCTGGGAGCCGTGAAGATG-3′	NM_001291065.1	62°C	100
	R:5′-GGCGTTCATGTTGCTCACGG-3′			
*Hgf*	F:5′- GGGATTCGCAGTACCCTCAC-3′ R:5′- TCGGATGTTTGGGTCAGTGG-3′	NM_001289458.1	60°C	134
*Fgf2*	F:5′- AACGGCGGCTTCTTCCTG-3′ R:5′- TGGCACACACTCCCTTGATAG-3′	NM_008006.2	60°C	133
*Fgf7*	F:5′- TCCTGCCAACTCTGCTCTAC-3′ R:5′- CTTTCACTTTGCCTCGTTTGTC-3′	NM_008008.4	59°C	234
*Fgf10*	F:5′- GTGCGGAGCTACAATCACCT-3′ R:5′- TCATTCTTGGTCCCGCTGAC-3′	NM_008002.4	60°C	113
*Igf1*	F:5′- GCTGGTGGATGCTCTTCAGT-3′ R:5′- TCCGGAAGCAACACTCATCC-3′	NM_010512.5	60°C	125
*Hoxa10*	F:5′- CTATCTGCTCCCTTCGCCAAA-3′ R:5′- CAAAAAGGAGTTCGCGGCAG-3′	NM_008263.3	60°C	73
*Calb1*	F:5′- TTGGCTCACGTCTTACCCAC-3′ R:5′- AAGCCGCTGTGGTCAGTATC-3′	NM_009788.4	60°C	119
*Vim*	F:5′-TGCTTCAAGACTCGGTGGAC−3′ R:5′-AAGCGCACCTTGTCGATGTA−3′	NM_011701.4	60°C	133
*Rpl19*	F:5′- TCATGGAGCACATCCACAAGCTGA-3′ R:5′- CGCTTTCGTGCTTCCTTGGTCTTA-3′	NM_009078.2	64°C	103

*Prl1a1, Prolactin family1, subfamily a, member 1, can deduce the rest of prolactin family members from this; Prlr, Prolactin receptor (long or short form); Pgr, Progesterone receptor; Esr1, Estrogen receptor 1 (alpha); Ki67, Proliferation marker protein Ki-67 gene; Foxa2, Forkhead box A2; Hgf, Hepatocyte growth factor; Fgf2, Fibroblast growth factor 2; Fgf7, Fibroblast growth factor 7; Fgf10, Fibroblast growth factor 10; Igf1, Insulin-like growth factor 1; Hoxa10, Homeobox A10; Calb1, Calbindin 1; Vim, Vimentin; Rpl19, Ribosomal protein L19*.

### Hematoxylin and Eosin Staining

4% PFA fixed uteri were dehydrated with gradient alcohol, transparent with xylene, and finally embedded in paraffin. Paraffin-embedded uteri were sectioned at a thickness of 5 μm. The tissue sections were placed on a baking table at 55°C for 1 h to melt the paraffin to facilitate the section fixation on the slide, then dewaxed by xylene, rehydrated by gradient alcohol, and stained with eosin and hematoxylin, dehydrated with gradient alcohol, transparent with xylene, Neutral gum sealed sheet.

### Statistical Analysis

All the experiments were independently repeated at least three times. Data were processed using GraphPad Prism 8 Software and shown as mean ± SEM. Normality was checked using the Shapiro-Wilk test. The significance of the difference between the two groups was assessed by Student's *t*-test. One- or two-way analysis of variance (ANOVA) test was used for the comparisons of multiple groups. Statistical significance was indicated by ^*^P < 0.05.

## Results

### Expression of Prolactin-Like Genes and Their Receptor in the Endometrium of Neonatal Mouse

**Figure 2:**
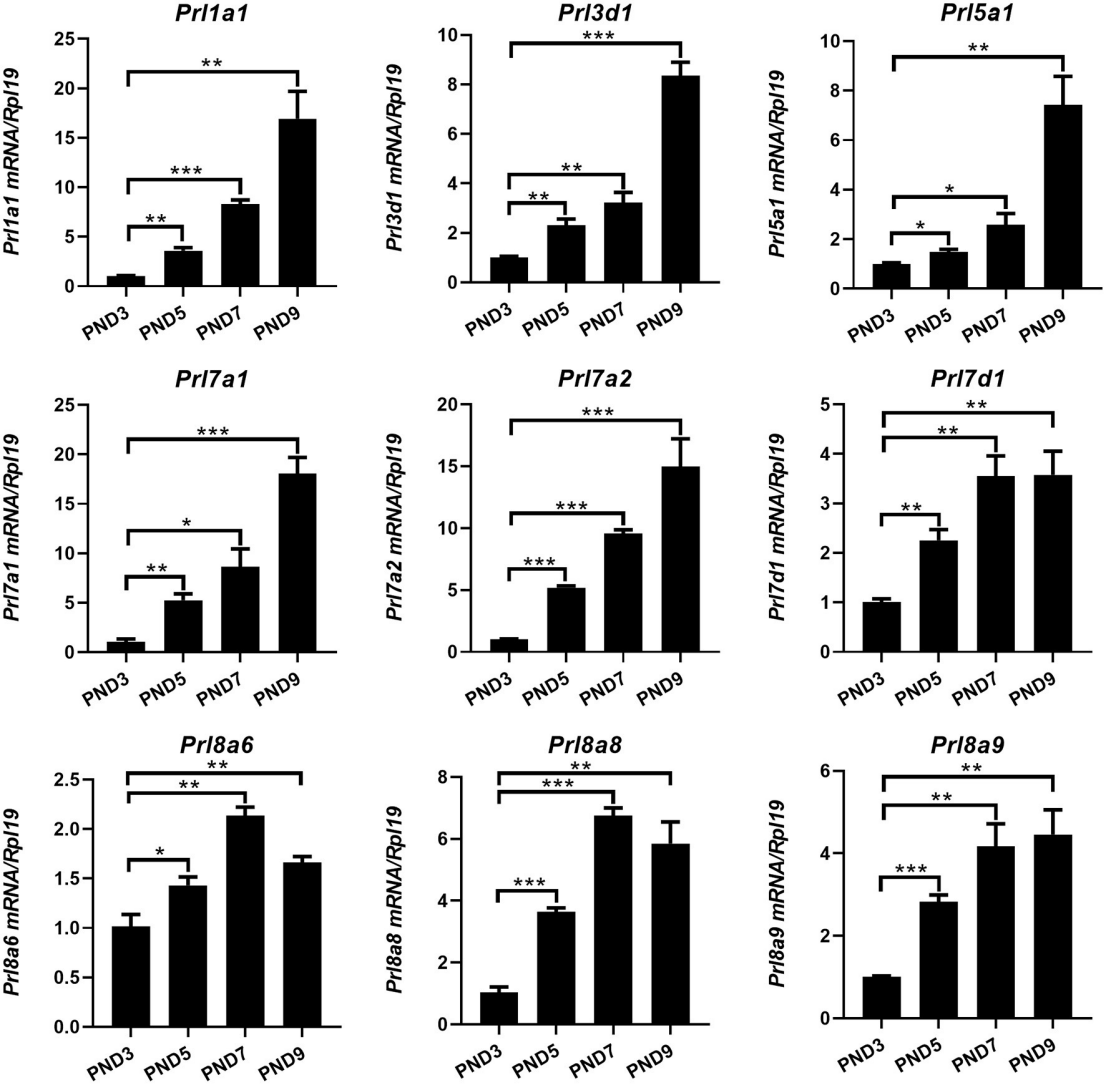
Quantitative Real-time-PCR analysis of *Prl1a1, Prl3d1, Prl5a1, Prl7a1, Prl7a2, Prl7d1, Prl8a6, Prl8a8*, and *Prl8a9* mRNA levels in the endometrium of neonatal mice during the early postpartum period. The mRNA expression level of 9 prolactin family members during the development of the uterine endometrium from PND3 to PND9.Results are means ± SEM (*n* = 5). Bars with different superscripts are significantly different (*P* < 0.05).

**Figure 3:**
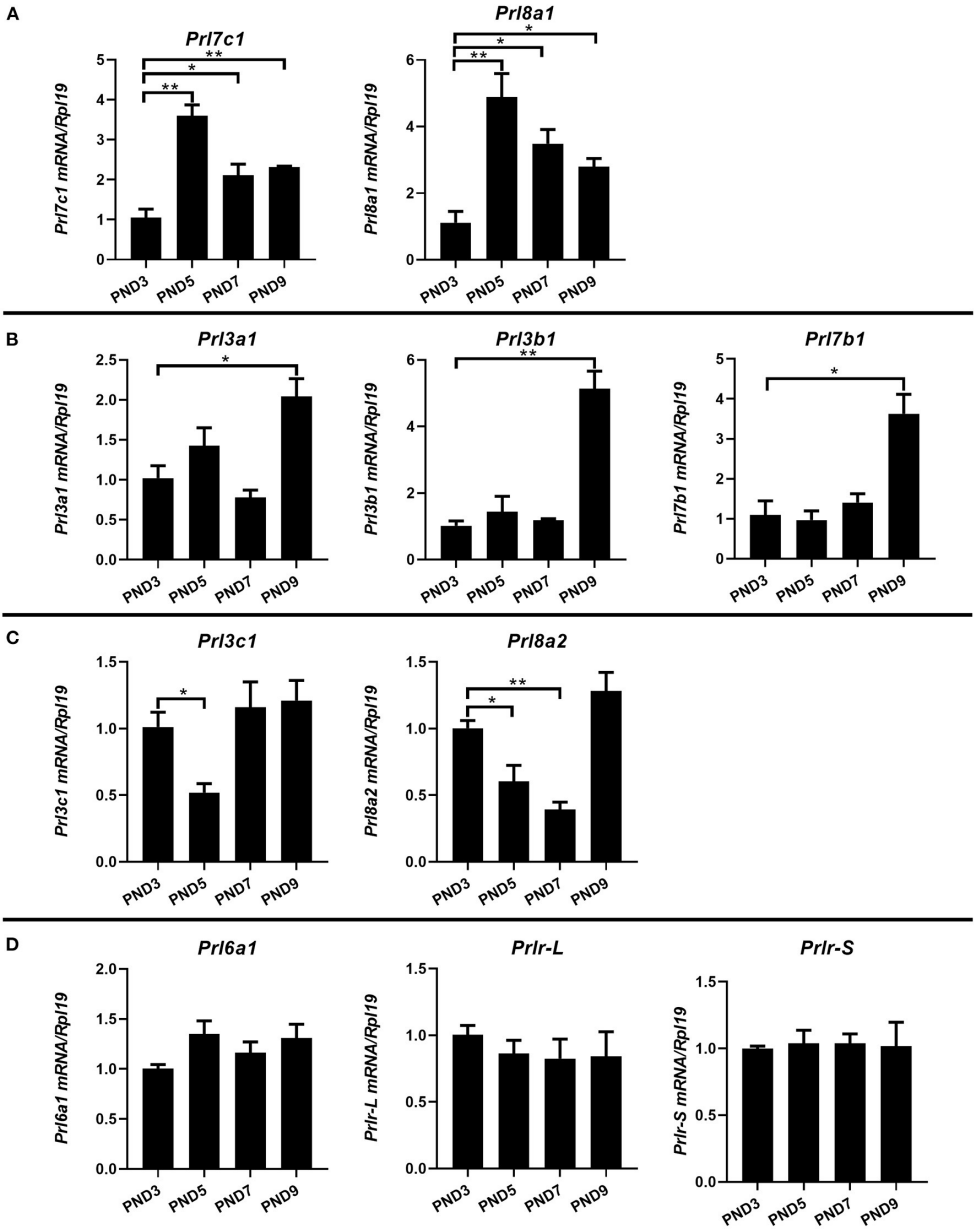
Quantitative Real-time-PCR analysis of *Prl3a1, Prl3b1, Prl3c1, Prl6a1, Prl7b1, Prl7c1, Prl8a1, Prl8a2, and Prlr* (*Prlr-L and Prlr-S*) mRNA levels in the endometrium of neonatal mice during the early postpartum period. The mRNA expression of *Prl7c1* and *Prl8a1* were up-regulated on PND5, and then down-regulated on PND7 and PND9 **(A)**. *Prl3a1, Prl3b1*, and *Prl7b1* genes increased on PND9 **(B)**. *Prl3c1* and *Prl8a2* genes decreased on PND5 **(C)**. The expression of *Prl6a1* and *Prlr* (*Prlr-L and Prlr-S*) were stable from PND3 to PND9 **(D)**. Results are means ± SEM (*n* = 5). Bars with different superscripts are significantly different (*P* < 0.05).

Different from humans in which only one prolactin gene is expressed, mice have 35 prolactin family members (PRLs). We detected the expression of PRLs' genes together with the prolactin receptor (PRL-R) in the endometrium of neonatal mice. Among the 21 prolactin family members we detected, 17 members could be detected in endometrium (*Prl1a1, Prl3a1, Prl3b1, Prl3c1, Prl3d1, Prl5a1, Prl6a1, Prl7a1, Prl7a2, Prl7b1, Prl7c1, Prl7d1, Prl8a1, Prl8a2, Prl8a6, Prl8a8*, and *Prl8a9*), while the remaining 4 PRLs were not detected (*Prl2a1, Prl2b1, Prl2c1* and *Prl4a1*). In mice, the critical period for endometrial glands' genesis and development are PND3 to PND9, especially PND7 to PND9. Among the 17 detectable PRLs, at three critical time points of glandular buds invagination (PND5), morphologic branching (PND7), and spiral curling (PND9) in mouse uterine glands, the expression of *Prl1a1, Prl3d1, Prl5a1, Prl7a1, Prl7a2, Prl7d1, Prl8a6, Prl8a8*, and *Prl8a9* genes were significantly increased, along with the development of uterine glands (Figure 2). *Prl7c1* and *Prl8a1* were observably up-regulated at uterine glandular buds invagination (PND5), and then slightly down-regulated on PND7 and PND9, but their expression levels were still stronger than those on PND3 (Figure 3A). Although *Prl3a1, Prl3b1*, and *Prl7b1* did not increase on PND5 and PND7, they suddenly increased significantly on PND9 (Figure 3B). Surprisingly, *Prl3c1* and *Prl8a2* were markedly down-regulated on PND5, and *Prl3c1* subsequently recovered to the expression level of PND3 on PND7 and PND9, while *Prl8a2* was still down-regulated on PND7, but also recovered to the expression level of PND3 on PND9 (Figure 3C). The expression of *Prl6a1* from PND3 to PND9 hardly changed. Meanwhile, we detected the receptors of the prolactin family members. The expression of *Prlr* (*Prlr-L and Prlr-S*) was extremely stable in the mice's endometrium after birth, and there was no significant change along with the development of uterine glands, but it was always highly expressed (Figure 3D).

### Expressions of Prolactin-Like Genes Were Inhibited by Progesterone Injection Associated With Abolished Adenogenesis of Mouse Uterus

Due to a large number of prolactin family members in mice, complete deletion of prolactin family members is very hard to achieve. Therefore, we changed the research strategy, reverted studied the relationship between prolactin and endometrial adenogenesis by inhibiting the generation of endometrial glands in neonatal mice using progesterone injection. Referring to previous studies, we conducted relevant tests to verify the success and feasibility of our model of endometrial gland genesis and developmental deletion in mice.

**Figure 4:**
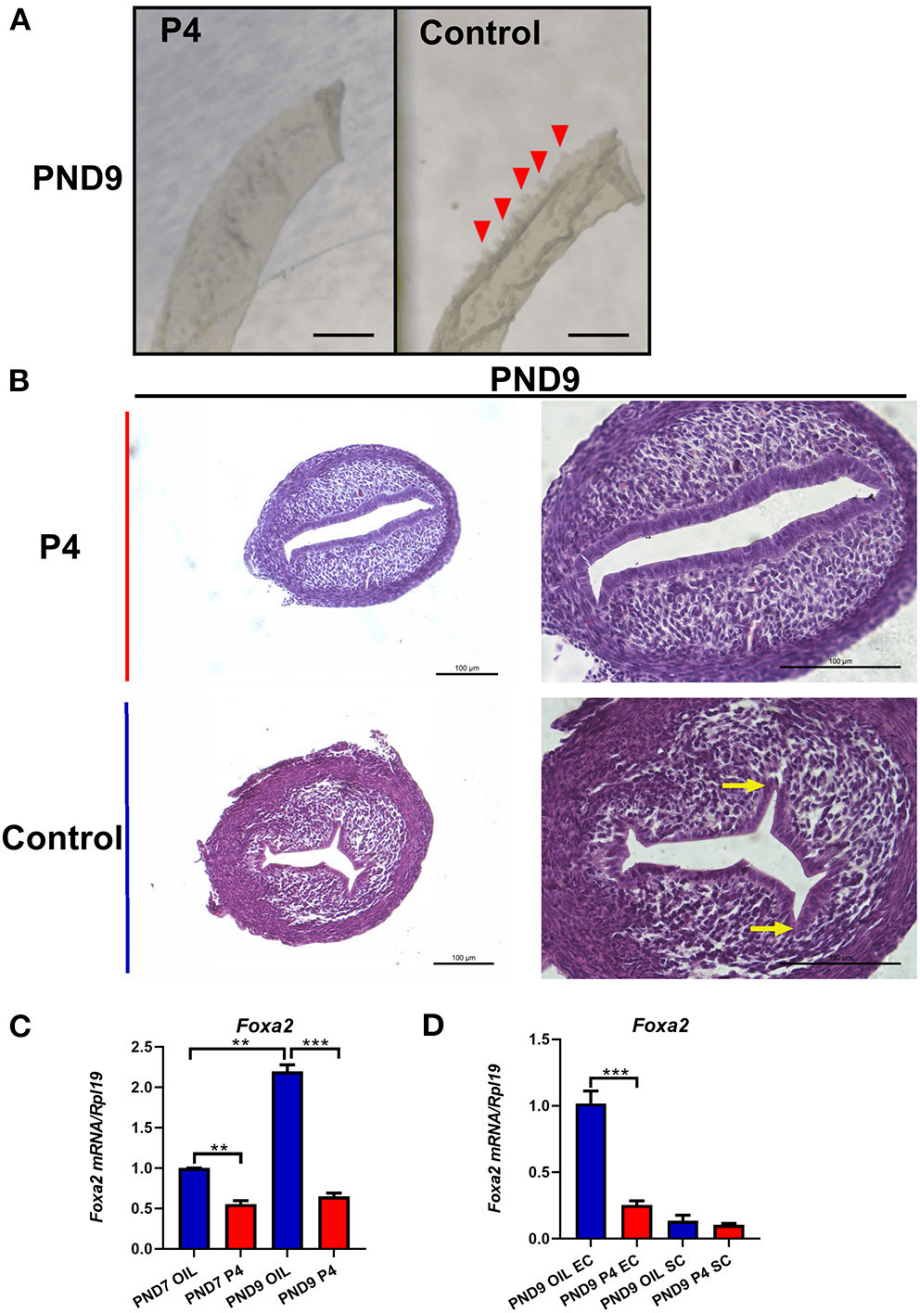
A successful model of endometrial adenogenesis and development loss was established. Representative pictures of endometrial epithelial morphology on PND9 after continuous subcutaneous injection of P4/Sesame oil of neonatal mice, the gland bud of the endometrial cavity indicated by the red triangle, Scale bar, 500 μm **(A)**. Hematoxylin and Eosin staining was performed on the uteri on PND9. The gland bud of the endometrial cavity is indicated by the yellow arrow, Scale bar, 100 μm **(B)**. The expression of *Foxa2* gene in whole uteri on PND7 or PND9 **(C)**, in epithelium cell (EC), or stroma cell (SC) on PND9 **(D)**. Results are means ± SEM (*n* = 5). Bars with different superscripts are significantly different (*P* < 0.05).

We observed epithelium which was isolated from the uterus of the neonatal mice and found that the P4 injected neonatal mice lacked the gland bud, while the control group which injected with sesame oil showed normal gland bud attached to the endometrial lumen (Figure 4A). After injection of P4, the budding phenomenon of uterine luminal epithelium branching into stroma was significantly reduced (Figure 4B). The mRNA level of *Foxa2*, a marker of endometrial glandular epithelium, was detected, and the results were consistent with the morphological results above. The whole uterus real-time quantitative PCR results showed that the expression of *Foxa2* on PND7 and PND9 was significantly down-regulated in the uterus of the P4 injected neonatal mice compared with the control group (Figure 4C). *Foxa2* expression was hardly detected in the stroma, and its expression was obviously inhibited in the uterine epithelium of the P4 injected neonatal mice (Figure 4D). These results indicate that injection of P4 can inhibit the generation and development of endometrial glands in neonatal mice, confirming the effectiveness and feasibility of this model.

**Figure 5:**
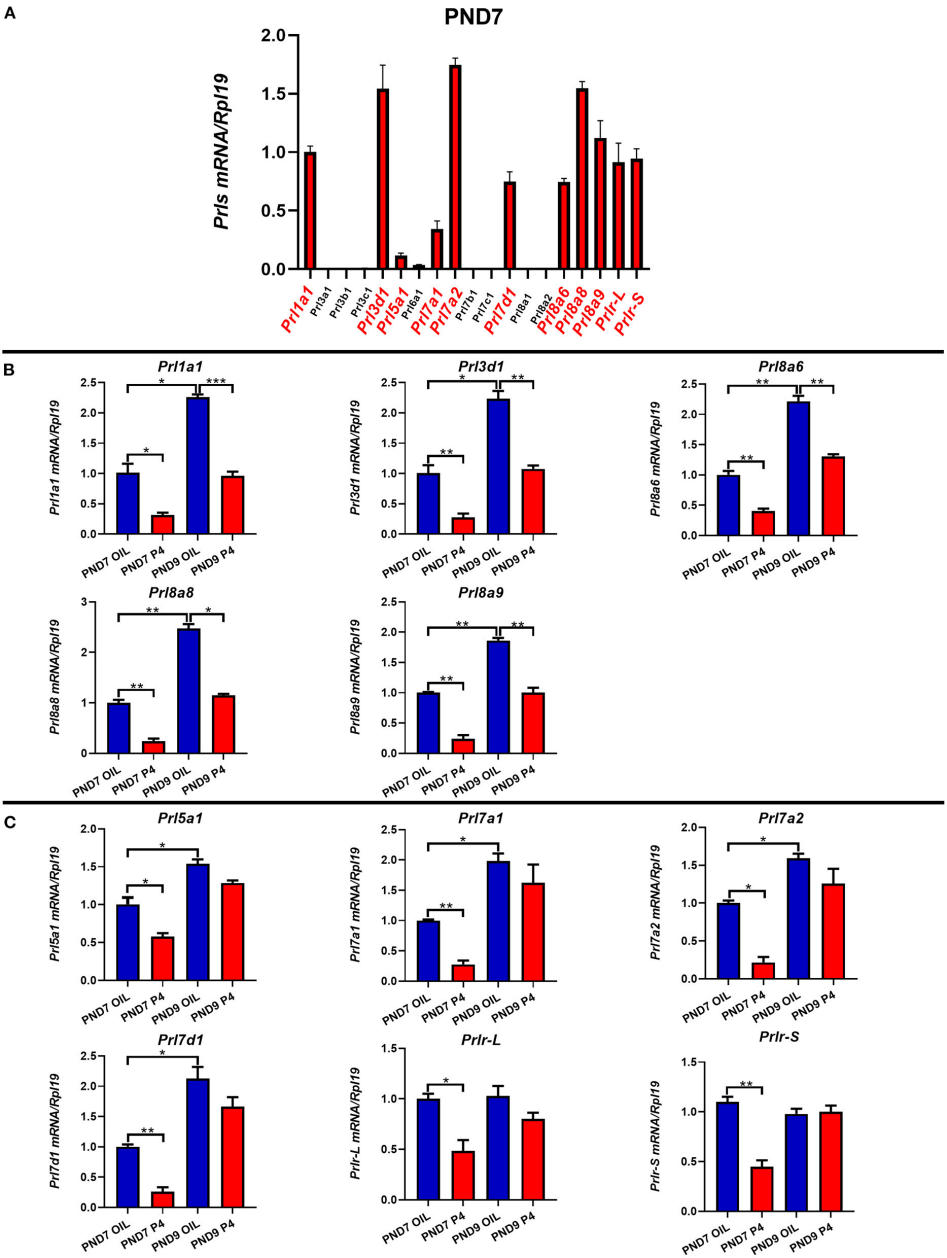
The expression of prolactin family members and prolactin receptor on PND7 and PND9 after treated with P4 or Sesame oil. Comparison of the prolactin family members and the prolactin receptor genes' expression on PND7 **(A)**. *Prl1a1, Prl3d1, Prl8a6, Prl8a8*, and *Prl8a9* genes were suppressed after endometrial adenogenesis inhibited by P4 injection on PND7 and PND9 **(B)**. The expression of *Prl5a1, Prl7a1, Prl7a2, Prl7d1*, and *Prlr* (*Prlr-L and Prlr-S*) were inhibited by P4 injection on PND7 **(C)**. Results are means ± SEM (*n* = 5). Bars with different superscripts are significantly different (*P* < 0.05).

To study the relationship between prolactin in the endometrium of mice and the generation and development of endometrial glands, the uterus was collected on PND7 and PND9, the key time points of the endometrial adenogenesis, and the expression of PRLs was detected on PND7 (Figure 5A). The results showed that *Prl1a1, Prl3d1, Prl5a1, Prl7a1, Prl7a2, Prl7d1, Prl8a6, Prl8a8, Prl8a9* were suppressed after adenogenesis inhibited by P4 injection on PND7 (Figures 5B,C). Furthermore, the expression level of *Prlr* was also significantly decreased by P4 injection on PND7. On PND9, *Prl1a1, Prl3d1, Prl8a6, Prl8a8*, and *Prl8a9* were still significantly inhibited, and their expression level was only equivalent to that of PND7 in neonatal mice injected with sesame oil (Figure 5B). However, the expression of *Prl5a1, Prl7a1, Prl7a2, Prl7d1*, and *Prlr* tended to be inhibited, but the degree of inhibition was reduced, with no significant statistical difference (Figure 5C).

**Figure 6:**
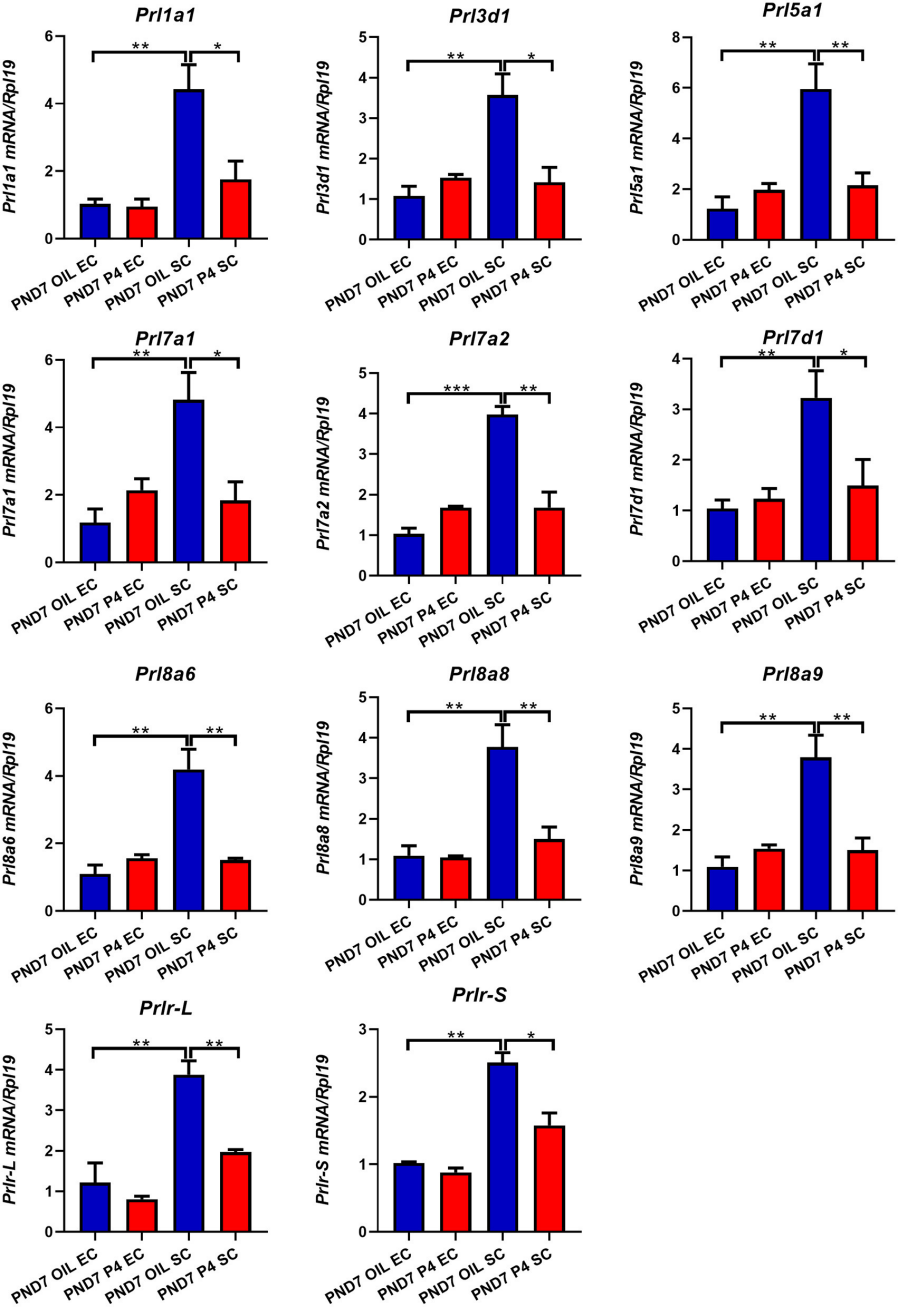
Quantitative Real-time-PCR analysis of *Prl1a1, Prl3d1, Prl5a1, Prl7a1, Prl7a2, Prl7d1, Prl8a6, Prl8a8, Prl8a9*, and *Prlr* (*Prlr-L and Prlr-S*) mRNA levels in epithelium or stroma on PND7. *Prl1a1, Prl3d1, Prl5a1, Prl7a1, Prl7a2, Prl7d1, Prl8a6, Prl8a8, Prl8a9, Prlr* (*Prlr-L and Prlr-S*) genes were mainly expressed in the stroma and inhibited after P4 injection on PND7. Results are means ± SEM (*n* = 5). Bars with different superscripts are significantly different (*P* < 0.05).

**Figure 7:**
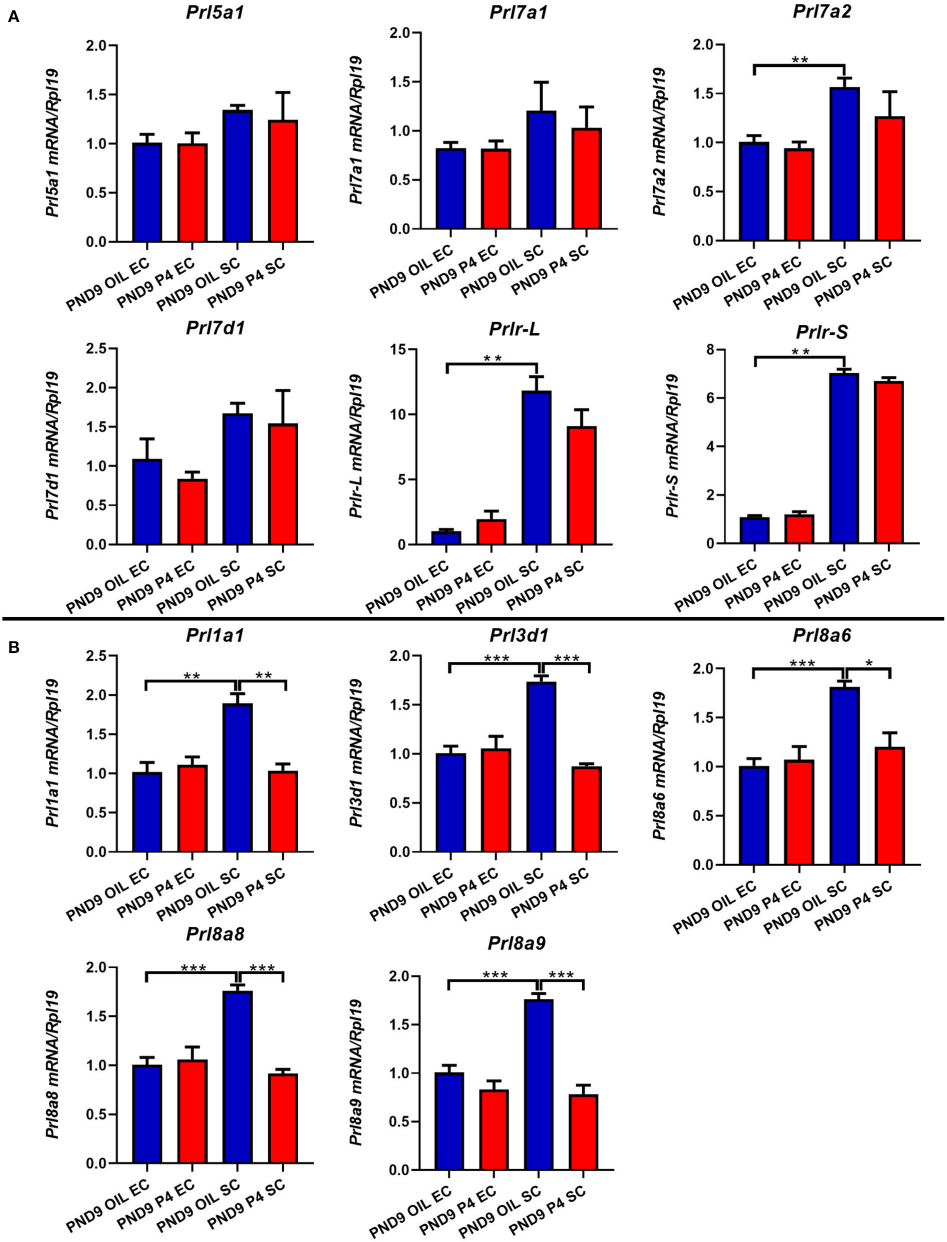
Quantitative Real-time-PCR analysis of *Prl1a1, Prl3d1, Prl5a1, Prl7a1, Prl7a2, Prl7d1, Prl8a6, Prl8a8, Prl8a9*, and *Prlr* (*Prlr-L and Prlr-S*) mRNA levels in epithelium or stroma on PND9. The expression level of *Prl5a1, Prl7a1, Prl7a2, Prl7d1*, and *Prlr* (*Prlr-L and Prlr-S*) returned to normal on PND9 **(A)**. *Prl1a1, Prl3d1, Prl8a6, Prl8a8*, and *Prl8a9* were significantly inhibited in the endometrial stroma on PND9 **(B)**. Results are means ± SEM (*n* = 5). Bars with different superscripts are significantly different (*P* < 0.05).

To further determine the specific expression location of prolactin family members in the endometrium, we separated the epithelium and stroma of the endometrium. We found that PRLs were expressed in both epithelium and stroma. However, on either PND7 or PND9, *Prl1a1, Prl3d1, Prl5a1, Prl7a1, Prl7a2, Prl7d1, Prl8a6, Prl8a8, Prl8a9*, and *Prlr* (*Prlr-L and Prlr-S*) were mainly expressed in the stroma, but not epithelium. Further careful comparison reveals that the expression of these nine prolactin family members and *Prlr* were inhibited in the endometrial stroma, but not significantly changed in the endometrial epithelium after P4 injection on PND7 (Figure 6). On PND9, the expression level of *Prl5a1, Prl7a1, Prl7a2, Prl7d1*, and *Prlr* returned to normal (Figure 7A), but *Prl1a1, Prl3d1, Prl8a6, Prl8a8*, and *Prl8a9* were still significantly inhibited in the endometrial stroma (Figure 7B).

### Association Among Endometrial Adenogenesis, Cell Proliferation, Growth Factors and the Expression of Steroid Hormones Receptors

**Figure 8:**
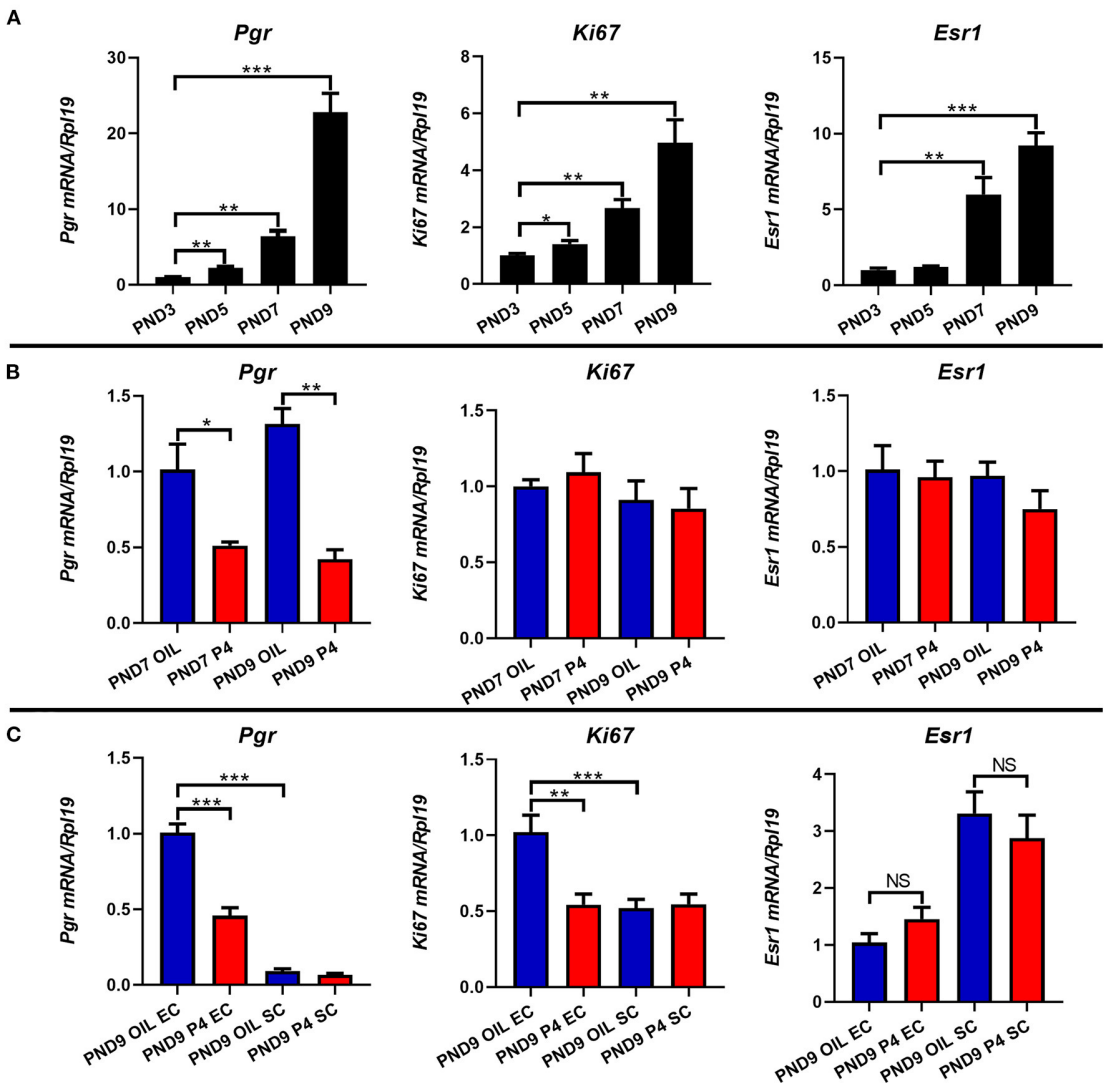
Effects of endometrial gland dysgenesis on the expression of cell proliferation, *Pgr, Esr1*. The expressions of *Ki67, Pgr*, and *Esr1* on PND3, PND5, PND7, and PND9 **(A)**. Comparison of *Ki67, Pgr*, and *Esr1* genes' expression after treated with P4 or Sesame oil on PND7 and PND9 **(B)**. The mRNA level of *Ki67, Pgr*, and *Esr1* in endometrial epithelium or stroma after treated with P4 or Sesame oil on PND9 **(C)**. Results are means ± SEM (*n* = 5). Bars with different superscripts are significantly different (*P* < 0.05).

It is well-known that estrogen and progesterone bind to their own receptors to regulate the morphology and function of the uterus. To investigate the association between endometrial adenogenesis, cell proliferation, and the expression of steroid hormone receptors, we examined the expression levels of cell proliferation marker (*Ki67*), estrogen receptor (*Esr1*), and progesterone receptor (*Pgr*) in the uterus of neonatal mice. The results showed that the expression of *Ki67, Esr1*, and *Pgr* increased gradually with the development of the uterus from PND3 to PND9 (Figure 8A). On PND7 and PND9, the expression of *Pgr* was significantly down-regulated when the endometrial adenogenesis was inhibited, while the expression of *Ki67* and *Esr1* was not significantly different (Figure 8B).

After separating the epithelium and stroma of the PND9 uterus, further analysis showed that *Pgr* was mainly expressed in epithelial cells, and when the endometrial adenogenesis was inhibited, *Pgr* in epithelial cells rather than stromal cells was significantly down-regulated. Meanwhile, the proliferation of epithelial cells (*Ki67*) was inhibited. *Esr1* was mainly expressed in the stroma, however, *Esr1* expression did not differ between the epithelium and stroma regardless of whether the occurrence of the uterus was inhibited (Figure 8C).

**Figure 9:**
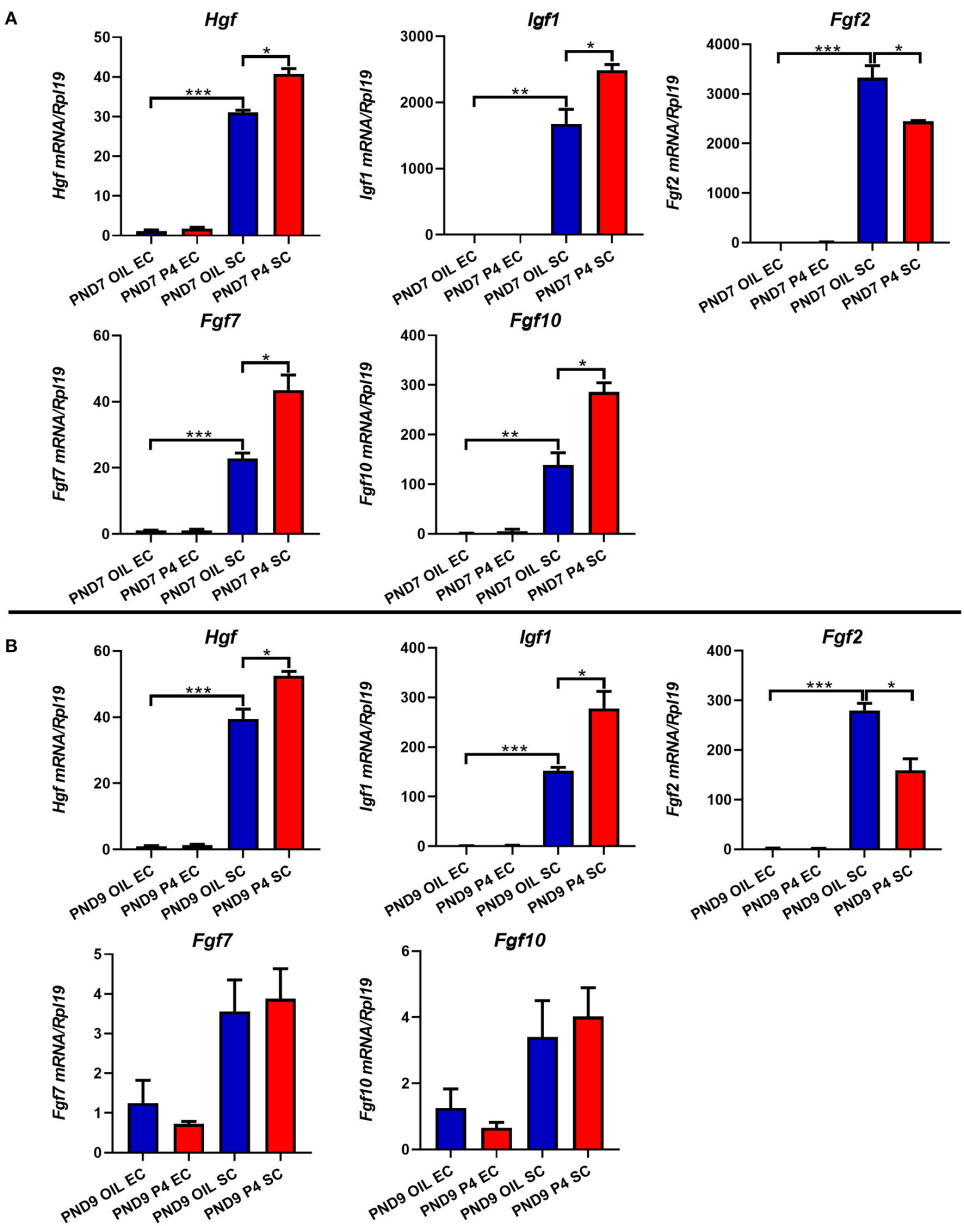
Effects of endometrial gland dysgenesis on the expression of *Hgf*, *Fgf2, Fgf7, Fgf10*, and *Igf1*. The expressions of *Hgf*, *Fgf2, Fgf7, Fgf10*, and *Igf1* in endometrial epithelium or stroma after treated with P4 or Sesame oil on PND7 **(A)** and PND9 **(B)**. Results are means ± SEM (*n* = 5). Bars with different superscripts are significantly different (*P* < 0.05).

In addition, multiple growth factors were detected. As shown in Figure 9, the expression levels of *Hgf*, *Fgf2, Fgf7, Fgf10*, and *Igf1* in the stroma were much higher than those in the epithelium. When the endometrial adenogenesis was inhibited, the expression levels of *Hgf* and *Igf1* in the stroma were significantly up-regulated on PND7 and PND9. *Fgf2*, in contrast, was down-regulated. The expression of *Fgf7* and *Fgf10* in the uterine stroma of the progesterone-treated group were up-regulated on PND7 but returned to the normal level on PND9.

## Discussion

Prolactin is a hormone/cytokine that participates in and coordinates a variety of biological processes in animals (19). In humans and other mammals like sheep, there is only one gene of the prolactin family in their genome. However, there are a large number of prolactin family member genes in mice (21). The role of each member in mouse uterine glandular genesis has not been studied. In this study, not all members of the prolactin family play a role in the uterus during adenogenesis in mice. Some of the family members, such as *Prl2a1, Prl2b1, Prl2c* (including *Prl2c1* to *Prl2c5*), and *Prl4a1*, were barely detectable in the uterus, indicating that they were not necessary during uterine development. Along with the development of the uterine glands, the expression levels of various prolactin family members are not consistent and synchronous, which indicates that the importance and function of various prolactin family members in the process of uterus development may be different. The expression of *Prl1a1, Prl3d1, Prl5a1, Prl7a1, Prl7a2, Prl7d1, Prl8a6, Prl8a8*, and *Prl8a9* genes gradually increased during uterine gland development. These prolactin family members are strongly expressed in the process of glandular buds invagination (PND5), morphologic branching (PND7), and spiral curling (PND9), indicating that these members may participate in and regulate the process of uterine development and endometrial adenogenesis. Our results are consistent with the prolactin expression pattern found by Ebling and Taylor et al. in neonatal ewes, in which serum PRL concentration is high at birth, increases from PND 1 to 14, and then decreases on PND 56 (30). Our and other‘s results suggest that prolactin expression is positively correlated with the onset of endometrial gland proliferation in the developing uterine wall (27, 31).

In addition, *Prl7c1* and *Prl8a1* are suddenly upregulated when uterine glandular buds invagination (PND5), suggesting that they may be essential key members in initiating and regulating endometrial adenogenesis. While *Prl3a1, Prl3b1*, and *Prl7b1* are highly expressed when uterine gland spiral curling occurs (PND9), may be helpful to promote the gland to continue to penetrate the stroma and further curl development. We also found that prolactin receptor expression was very stable and was strongly expressed at PND3, suggesting that prolactin receptor is involved in mouse uterine development from beginning to end and plays an important and stable role in it, and maybe necessary. The stable expression of prolactin receptors also further supports and reveals the theory that members of the prolactin family are essential in mouse uterine development and endometrial adenogenesis.

Our study also found that the prolactin family members were mainly expressed in the endometrial stroma, and the inhibition of prolactin family members' expression also occurs in the endometrial stroma. Studies have shown that the development and function of the uterus depend on the interaction between epithelial cells and stromal cells (32). These interactions play an important role in uterine morphogenesis and the control and coordination of a variety of important cellular behaviors, including movement, adhesion, differentiation, and proliferation (33). Endometrial morphogenesis and the establishment of normal uterine tissue structure require interaction between epithelium and stroma (34). In some epithelial-stromal organs, prolactin promotes and stimulates epithelial differentiation and development in a synergistic manner with extracellular stroma signaling (17). At the same time, extracellular stroma can influence the pattern of branching morphogenesis by controlling the cell cycle, apoptosis, and the expression of stroma and epithelial development genes (35, 36). Our results reveal that multiple PRLs are involved in uterine development and endometrial adenogenesis, with multiple members playing important roles in this process. Continuous progesterone treatment may alter the expression pattern of prolactin in endometrial stromal cells, thereby altering the composition of the extracellular matrix, affecting the autosecretory and paracrine functions of stromal cells, and stromal-epithelial interaction and communication. It has been shown that hepatocyte growth factor (HGF) and fibroblast growth factors (FGFs) signaling pathways may regulate uterine growth and development as paracrine and/or autocrine mediators of epithelial-stromal interactions (37–39). The Insulin-like growth factor (IGF) system in the sheep uterus regulates the epithelial-stromal interaction, which is important for postnatal uterine growth and endometrial gland morphogenesis. We found that PRLs synergistically interact with multiple growth factors (*Hgf*, *Fgf2, Fgf7, Fgf10*, and *Igf1*) to regulate the differentiation and development of luminal epithelium and ultimately generate endometrial glands through the epithelial-stroma interaction.

In the uterus, E2 and P4 exert their functions mainly by binding their nuclear receptors ER and PR, which play an indispensable role in regulating uterine development and function (40, 41). In our study, the expression of *Esr1* and *Pgr* gradually increased with the development of the uterus, and *Esr1* was mainly expressed in the stroma while *Pgr* was mainly expressed in the epithelium. When continuous injection of P4 inhibits the occurrence and development of endometrial glands of mice, the expression of the prolactin family members was a certain degree of inhibition, and the expression of *Pgr* was also significantly down-regulated, but the expression of *Esr1* did not change, suggesting that *Pgr* plays a more important role in the development of mouse uterus glands, and prolactin family members are closely related to *Pgr*, and they may exist interaction, mutual adjust the development of the uterus.

In the uterus of ruminant animals, especially bovine and ovine, PRL plays a role in establishing and maintaining pregnancy, promoting the development and secretion of uterine glands, and providing an appropriate environment for embryo implantation (42, 43). Members of the prolactin family play an important role in the formation and development of endometrial glands and also play multiple key roles in pregnancy. The absence of any member of the prolactin family in mice can lead to pregnancy failure. Previous studies have shown that *Prl*^−/−^ systemic knockout of *Prl* can affect the estrous cycle, leading to abnormal mammary gland development and infertility in female mice (24). *Prl7d1*^−/−^ knockout can lead to the thickening of the placental decidual spiral artery, resulting in reduced litter size and decreased fertility (25, 44). *Prlr*^−/−^ knockout can lead to female infertility and failure of progesterone production by the corpus luteum of the ovary in mice, leading to pregnancy failure (45). However, it has also been found that systemic knockout of *Prl4a1, Prl7b1, Prl8a2*, and other members of the prolactin family does not cause pregnancy failure (13–15). Therefore, the prolactin family members are important for uterine development and pregnancy in mice, not every member but only a few important members are indispensable. In our study, *Prl1a1, Prl3d*1, *Prl8a6, Prl8a8*,and *Prl8a9*, and other prolactin members are involved in the process of uterine development and endometrial adenogenesis. When endometrial adenogenesis is inhibited, their expressions are also inhibited. Therefore, in future studies, researchers may carefully consider and construct prolactin gene knockout engineered mice to further explore the relationship between prolactin and uterine development and endometrial adenogenesis, as well as the functional role of prolactin family members in mouse uterus.

A large number of studies have shown that hyperprolactinemia can cause endometrial glandular hyperplasia and even endometrial adenomyosis in adult mice, rabbits, and pigs, (46–48) while hyperprolactinemia can cause endometrial glandular hyperplasia in neonatal sheep uterus (27). Therefore, high expression of prolactin promotes endometrium proliferation (49). Even though the formation and development of endometrial glands in mice are inhibited, and the expression of some prolactin family members (*Prl5a1, Prl7a1, Prl7a2, Prl7d1*) is inhibited on PND7, the expression level of these members are soon restored to an almost normal level by PND9. This suggests that there may be some potential regulatory mechanisms in the uterus. When the endometrial adenogenesis is inhibited, it is attempted to enhance the proliferation of endometrial glands by increasing the expression level of prolactin family members, to restore the endometrial adenogenesis. However, the regulatory mechanism involved is still unknown and needs further exploration and research.

In conclusion, members of the mouse prolactin family are closely associated with uterine development and endometrial adenogenesis. Multiple members play roles in this process and are indispensable. Prolactin family members interact with estrogen receptors and progesterone receptors and cooperate with various growth factors to regulate the development of the mouse uterus. Prolactin family members not only participate in the development of uterine morphology but also play a key role in maintaining normal pregnancy in mice. However, unfortunately, due to the lack of commercial antibodies for prolactin-related proteins, only mRNA levels of these genes can be obtained so far in this study, and these research results are just a few, and more profound secrets await further investigation.

## Data Availability Statement

The original contributions presented in the study are included in the article/Supplementary Material, further inquiries can be directed to the corresponding author.

## Ethics Statement

The animal study was reviewed and approved by the Animal Care and Use Committee of South China Agricultural University.

## Author Contributions

RS conceived the study. RS and JK wrote the paper. JK, YL, YZ, WY, and YW performed experiments. RS and JK analyzed data. All authors contributed to the article and approved the submitted version.

## Funding

This study was funded by the National Key Research and Development Program of China (Grant/Award No. 2018YFC1004400) and the National Natural Science Foundation of China (Grant/Award Nos. 31900601 and 31771664).

## Conflict of Interest

The authors declare that the research was conducted in the absence of any commercial or financial relationships that could be construed as a potential conflict of interest.

## Publisher's Note

All claims expressed in this article are solely those of the authors and do not necessarily represent those of their affiliated organizations, or those of the publisher, the editors and the reviewers. Any product that may be evaluated in this article, or claim that may be made by its manufacturer, is not guaranteed or endorsed by the publisher.

## Abbreviations

EDTA: Ethylene diamine tetraacetic acid
FBS: Fetal bovine serum
GE: glandular epithelium
HE: Hematoxylin and Eosin staining
LE: luminal epithelium
LIF: Leukemia inhibitory factor
P4: Progesterone
PCR: Polymerase chain reaction
PFA: Paraformaldehyde
PND: Postnatal day
PRL-R: Prolactin receptor
PRLs: Prolactin family members.
