# Supplementary material for: The Influence of the Prolactins on the Development of the Uterus in Neonatal Mice
Source: Front Vet Sci. 2022 Feb 17;9:818827. doi: 10.3389/fvets.2022.818827 (PMC8891943; doi:10.3389/fvets.2022.818827)
Supplement: Supplementary file 1 [file Image_1.pdf]

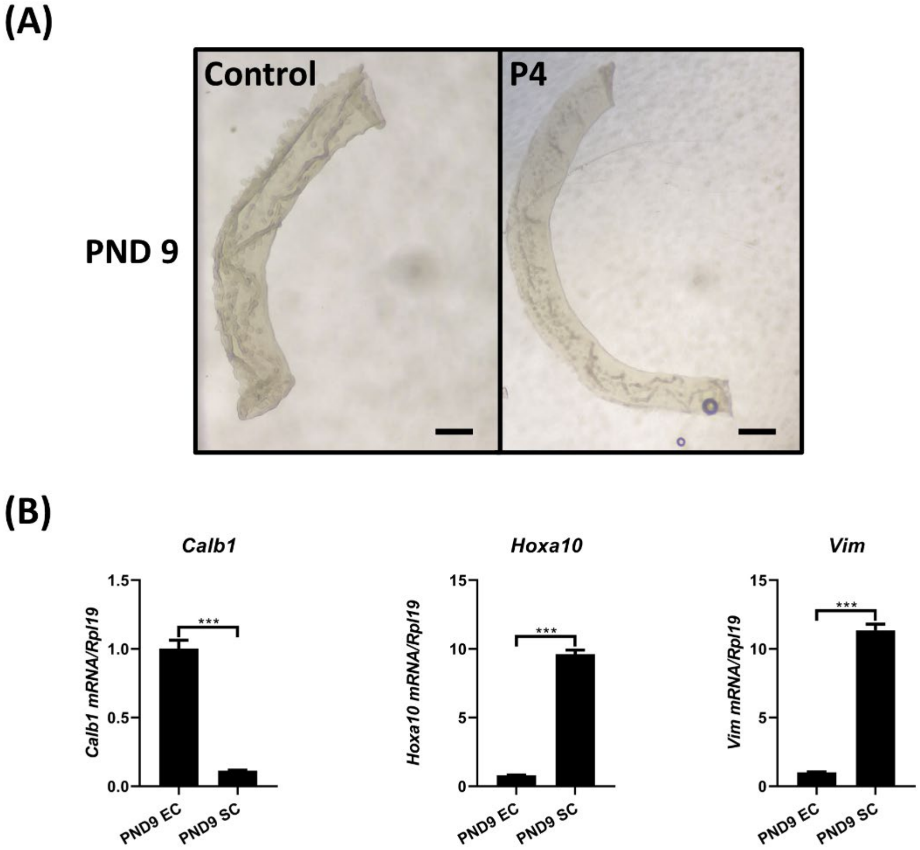

Figure S1 Intact uterine epithelium was digested and isolated from neonatal mice and its purity verification. Representative pictures of Endometrial epithelium digested and isolated from neonatal mice, Scale bar, 500 μm (A). The expression of epithelial cell marker *Calbindin 1* (*Calb1*) and stromal cell marker *Homeobox A10* (*Hoxa10*) and *Vimentin* (*Vim*) in EC or SC on PND9 (B). EC, epithelium cell; SC, stroma cell. Results are means ± SEM (n = 5). Bars with different superscripts are significantly different ($P < 0.05$).
